# Group meta-cognitive therapy and depression in women with breast cancer: a randomized controlled trial

**DOI:** 10.1186/s12905-021-01258-9

**Published:** 2021-03-18

**Authors:** Elham Zahedian, Masoud Bahreini, Nezamaddin Ghasemi, Kamran Mirzaei

**Affiliations:** 1 Nursing and Midwifery School, Bushehr University of Medical Sciences, Bushehr, Iran; 2 Department of Psychology, Salman Farsi University of Kazerun, Kazerun, Iran; 3 Community Medicine, Medical School, Bushehr University of Medical Sciences, Bushehr, Iran

**Keywords:** Breast cancer, Depression, Cognitive emotion regulation, Meta-cognitive therapy, Metacognitive beliefs

## Abstract

**Background:**

Breast cancer is one of the most common cancers in Iranian women. They will experience a mental health problem like depression before, during or after treatment. This study aimed to determine the effectiveness of group metacognitive therapy on depression, cognitive-emotional regulation, and meta-cognitive beliefs in women with breast cancer.

**Methods:**

In this randomized controlled clinical trial, a total of 24 depressed patients with breast cancer were randomly allocated to experimental and control groups. The experimental group received meta-cognitive therapy in 8 weekly sessions, but the control group received treatment as usual. Beck Depressive Inventory, cognitive emotion regulation questionnaire, and meta-cognitions questionnaire were completed before, after and one month after the intervention. Data were analyzed using Wilcoxon and Chi-square tests.

**Results:**

The mean score of depression in the experimental group was reduced from 21.6 ± 4.83 in the pre-test to 13.83 ± 8.12 in one-month follow-up (*p* = 0.16); however, there was no significant difference in the control group. The mean score of cognitive emotion regulation did not show a significant change in the two groups during the study and follow-up period. The mean score of meta-cognitive beliefs reached 68.75 ± 15.74 from 79.51 ± 10.72 in the experimental group during the follow-up period (*p* = 0.006); however, there was no significant difference in the control group in the score of metacognitive beliefs.

**Conclusion:**

These findings support the efficacy of meta-cognitive therapy as a viable psychosocial intervention in depressed patients with breast cancer.

*Trial registration* IRCT201606288473N5. Registered on: 05/09/2016 https://www.irct.ir/trial/8946.

**Supplementary Information:**

The online version contains supplementary material available at 10.1186/s12905-021-01258-9.

## Background

Breast cancer is the most common cancer among women [1] and is the second cause of cancer death after lung cancer [2]. In the United States, more than 3.8 million women with a history of breast cancer were alive on January 1, 2019 [3]. Some of these women were cancer-free, while others still had evidence of cancer and might have undergone treatment [4]. In Iran, breast cancer is the most common type of cancer in women, which is ranked the fifth in cancer mortality. The age of the onset of the disease in Iranian women is a decade earlier than those in other developed countries [5]. Depression is one of the most common psychiatric disorders among women with breast cancer [6]. Results of some studies indicated that 58% of patients with cancer had mild depression and 38% had major depression [7]. Depression, reduced energy, and increased stress from the disease can ultimately lead to a reduction in immune function, prolonged recovery time, or a decline in the quality of life of patients with breast cancer [8]. These symptoms may also reduce individual and social performance [9]. Results of some studies have shown that an increase in life expectancy in these patients is associated with psychological interventions [10].

In the last few years, Meta-Cognitive Therapy (MCT) has been considered a novel approach to address various mental disorders. Findings indicated that metacognition appeared to be linked to anxiety, depression, and quality of life in patients with medical chronic conditions. Therefore, dysfunctional metacognitive beliefs might be a relevant factor associated with the process of adapting to illness [11]. Researchers believe that negative thoughts are the pivotal factor in depression disorder, so that negative attitudes are the main component of mood change [12]. Moreover, changes in each of the different sections of the cognitive system, such as memory, attention, and consciousness, make changes in the mood. In this approach, emotional disorders are attributed to the defect in controlling the negative emotions of negative thoughts and beliefs about the concern and the lack of using effective methods to counteract negative emotions. Consequently, the cognitive-emotional regulation in the adaptation of individuals with stressful life events plays a crucial role that cannot be ignored [13].

The MCT is based on the Self-Regulatory Executive Function Model proposed by Wells and Matthews [14]. Researchers Defined meta-cognition as “Awareness and management of one’s own thought”. Meta-cognition is generally referred to the second order cognition, that is, thoughts about thoughts or knowledge about knowledge [15]. According to the fundamental bases of this therapy, the psychological disorder occurs when a maladaptive thinking style called the Cognitive Attention Syndrome (CAS) is activated. This syndrome has an important effect on emotional regulation. Therefore, the purpose of the meta-cognitive model is to treat this syndrome and adjust the related meta-cognitive beliefs [16].

Meta-cognitive beliefs are also an important component in coping with depression. Meta-cognitive beliefs are, in fact, conscious assessments and interpretations from the concept of meta-cognitive thoughts and feelings in the individual and judgment about his\her cognitive status. Therefore, it can be stated that meta-cognitive experiences can correlate with emotional disorders [16]. Meta-cognitive beliefs include two categories of negative and positive beliefs. Negative beliefs are referred to as beliefs concerned with the uncontrollability of meaning and meta-cognitive experiences. In addition, the importance and danger of thoughts and positive beliefs are associated with benefits of cognitive activities, such as thought rumination and concern [17].

A growing body of research on MCT indicates an increase in the acceptance of this intervention. Bahrami et al. (2015) conducted a semi-experimental study to evaluate the effectiveness of meta-cognitive group therapy on meta-cognitive beliefs in women with cancer. The results demonstrated that meta-cognitive therapy had a significant effect on the reduction of symptoms associated with meta-cognitive factors in women with breast cancer [18]. There is increasing evidence to support the clinical use of MCT for the treatment of depression. Data from randomized trials of anxiety and depression have shown recovery rates in the MCT of 72–80% [19]. In a randomized trial study, researchers compared MCT to the gold standard treatment, CBT, in patients with GAD. According to the results of this study, researchers concluded that MCT appeared to produce recovery rates exceeding those of CBT [20]. Normann and Morina in a systematic review provided an updated meta-analytic review of the effect of MCT on psychological complaints. Their findings indicate that MCT is an effective treatment for various psychological complaints. They maintained that to date, the strongest evidence exists for anxiety and depression [21].

As mentioned, the chronic and prolonged nature of breast cancer disease causes it to be very difficult for patients to accept and cope with it. Depression is one of the most common psychiatric disorders associated with a breast cancer diagnosis. Furthermore, studies suggest that depression affects cognitive and meta-cognitive styles as well as adaptive methods of regulating emotions in individuals. Therefore, the third generation of cognitive-behavioral psychotherapies like meta-cognitive therapy seeks to modify individuals’ mental processes.

### Aim of the study

The aim of the study was to determine the effectiveness of Group MCT on depression, cognitive-emotional regulation, and meta-cognitive beliefs in women with breast cancer.

## Methods

### Design and setting

This two-armed controlled clinical trial study was conducted in Shiraz in southern Iran. The study protocol was written in accordance with the CONSORT guidelines [22]. This study was conducted in the Breast Disease Research Center that is one of the largest breast disease research and treatment centers in southern Iran, which has annually over 25,000 visitors from different parts of the country.

### Eligible criteria

The study participants were recruited from women with breast cancer who referred to the Breast Disease Research Center. The inclusion criteria admitted (a) adults 20–70 years of age, (b) being treated for a diagnosis of breast cancer, (c) mild to moderate depression score by the Beck Depressive Inventory and (d) the ability to attend sessions according to the oncologist. The exclusion criteria were absence from treatment sessions, death, and severe psychological or physiological conditions coinciding with the period of psychotherapy.

Estimation of sample size was set at 12 participants in each treatment arm, assuming a standard difference of 1.2 points on the primary outcome, and considering a bilateral significance level of 5% and a statistical power of 85%, adjusted by a dropout rate of 20%. The estimation of sample size was based on a study by researchers who reported the mean and standard deviation of depression in control and intervention groups 21 ± 1.4 and 19.50 ± 1.5, respectively [23].

### Measurements

At baseline, the patients’ demographic data (age, marital status, occupational status, educational status, place of residence, history of depression, history of hospitalization, duration of cancer, type of cancer therapy, history of psychotherapy and frequency of fertility) were evaluated using a demographic questionnaire.

### Primary outcome

The primary outcome measure was changed in depression score, and measured at baseline (T0), immediately after (T1), and one month after the intervention (T2) using the Beck Depressive Inventory (BDI). The BDI is a 21-item self-report rating inventory measuring characteristic attitudes and symptoms of depression. The questionnaire is designed for adults and adolescents over 13 years old. The person is asked to consider his or her feelings in the last two weeks and answer the questions. The 4-point Likert scale is from 0 to 3 for each question. This scale determines the degree of depression from mild to very severe. The score ranges from at least zero to at most 63. Internal consistency for the BDI ranges from 0.73 to 0.92 with a mean of 0.86. The BDI demonstrates high internal consistency, with alpha coefficients of 0.86 and 0.81 for psychiatric and non-psychiatric populations, respectively [24]. The BDI-Persian had high internal consistency (Cronbach’s alpha = 0.87) and acceptable test–retest reliability (r = 0.74) [25]. The BDI takes approximately 10 min to complete (see Additional file 1).

### Secondary outcomes

#### Cognitive emotion regulation questionnaire (CERQ)

This 36-item questionnaire evaluates the cognitive strategies that each person uses after experiencing threatening events or life stresses. This scale consists of the following 9 sub-scales, each consisting of four items and each referring to what someone thinks after experiencing threatening or stressful life events: self-blame, other-blame, rumination, catastrophizing, putting into perspective, positive refocusing, positive reappraisal, acceptance, and planning. Cognitive emotion regulation strategies were measured on a 5-point Likert scale ranging from 1 (almost never) to 5 (almost always). Individual subscale scores were obtained by summing the scores belonging to the particular subscale (ranging from 4 to 20). The total score is placed in the range of 36–180. The psychometric properties of the CERQ were studied in an adult general population sample. The results indicated that the CERQ had good factorial validity and high reliability, with Cronbach’s alpha ranging from 0.75 to 0.87 [26]. This questionnaire is a valid, reliable, and appropriate instrument to detect and assess cognitive strategies in Iranian patients [27] (see Additional file 2).

#### Meta cognitions questionnaire (MCQ)

The MCQ-30 is a short version of the original MCQ measuring individual differences in a selection of meta-cognitive beliefs, and judgments as well as monitoring tendencies considered important in the meta-cognitive model of psychological disorders. The questionnaire is 30-item, 4-point Likert, and self-report scale. The questionnaire has five subscales. The five subscales of the MCQ-30 are: cognitive confidence, positive beliefs in worry, cognitive self-consciousness, negative beliefs in the uncontrollability of thoughts and danger, and beliefs in the need to control thoughts. Subscale scores range from 6 to 24, and total scores range from 30 to 120, with higher scores indicating higher levels of unhelpful metacognitions. Subscales are calculated by summing the following item: lack of cognitive confidence: 8, 14, 17, 24, 26 and 29; positive beliefs in worry: 1, 7, 10, 19, 23 and 28; cognitive self-consciousness: 3, 5, 12, 16, 18 and 30; negative beliefs in uncontrollability and danger: 2, 4, 9, 11, 15 and 21; need to control thoughts: 6, 13, 20, 22, 25 and 27. The MCQ-30 exhibited good internal consistency, convergent validity, and acceptable to good test–retest reliability [28]. The Cronbach's alpha coefficient of the subscales varied from 0.72 to 0.93. The Cronbach's alpha coefficient of the total scale in the Iranian sample has been reported as 0.91 [29] (see Additional file 3).

### Blindness and randomization

Owing to the nature of meta-cognitive therapy, neither the therapist nor the participants can be blinded to the delivered treatment. Data collectors and data analyzers were blind to group membership.

Participants were randomized on a 1:1 basis into the intervention group (meta-cognitive therapy) or the control group (Treatment As Usual; TAU). To randomly allocate the participants to one of the two groups (intervention and control), a computer-generated random number sequence (https://www.randomizer.org/) was used by means of a simple allocation strategy. A statistician not involved in the study performed the randomization.

### Interventions

This study was conducted in the fall and winter of 2017. The participants were recruited from patients with breast cancer who referred to the Breast Cancer Disease Research Center. Patients with breast cancer who had mild to moderate depression, were enrolled as participants based on the Beck Depressive Inventory. After obtaining informed consent, samples provided their phone numbers and addresses to participate and announce the time, date and place of the therapy sessions by phone (education place was at the Breast Disease Research Center). The samples were randomly allocated to two experimental and control groups. Initially, the pretest (MCQ-30 and CERQ) was conducted to obtain the basic data. Then, eight 90-min sessions were held for the intervention group in addition to TAU (Table 1). Each week, a session was held in groups with assignments during and between sessions for this group of samples. The control group received treatment as usual. TAU consisted of providing the usual treatment to patients according to accepted standards for depression (except psychotherapy, which will not be allowed 6 months before the study or during it). Post-test was conducted after completing the sessions and one month later from both groups. The method of teaching was a lecture, group discussion, role play, and homework according to Welles' educational program [30].

**Table 1:** Content of MCT

Session	Content	Time (min)
1	Welcome, provide summaries of the treatment model and goals of the sessions, case formulation, conduct a pre-test, practice ATT, complete the ATT summary sheet Homework: Practice the mindfulness training technique (twice a day), record daily practice the mindfulness training technique	90
2	Review the homework, socialize the patient to the maintaining processes, including the impact of worry and rumination and the ineffectiveness of current coping strategies, review negative beliefs and rumination as ineffective coping strategies, introduce and practice Detached Mindfulness (DM), introduce thinking procrastination, as an experiment to change beliefs related to thinking uncontrollability, practice ATT Homework: Practice ATT, apply DM and thinking delay	90
3	Review of Homework and MDD-S Scale, review of CAS, metacognitive beliefs are verbally challenged in Socratic dialogues, and behavioral experiments are used to test and generate change in the person's metacognitive predictions of or beliefs in CAS strategies, instruct to postpone worry and rumination processes, identify thinking motivators, and apply DM, practice ATT Homework: Record thoughts, practice ATT, use DM, schedule activity Postpone thinking, increase the level of activity	90
4	Review the homework and MDD-S scale, negative beliefs related to thinking uncontrollability, levels of activity and maladaptive coping, check the use of thought postpone, challenges with positive metacognitive beliefs related to thinking, practice ATT Homework: Record thoughts, practice ATT, expand the application of DM, procrastination, schedule activity	90
5	Review the homework and complete the MDD-S scale, positive metacognitive beliefs, levels of activity and dysfunctional coping, check the continuous and widespread use of DM, continue to challenge positive metacognitive beliefs related to thinking, review the levels of activity and suggestions for improving it (identifying and stopping other ineffective coping techniques such as sleeping too much and drinking alcohol), practice ATT Homework: Practice ATT, postpone thinking, increase the activity level	90
6	Review the homework and MDD-s scale, positive metacognitive beliefs, activity levels, examine and challenge negative beliefs about emotion and depression, practice ATT (increase the difficulty level) Homework: Practice ATT, postpone thinking, continue activities	90
7	Review the homework and the MDD-S scale, metacognitive beliefs and dysfunctional coping, work on developing a new processing plan (complete the program summary sheet and provide a copy to the patient), review and overcome fears the patient recovers from depressive symptoms, practice ATT Homework: Practice ATT, apply and reinforce	90
8	New processing plan, start work on the initial treatment plan, review homework and MDD-S scale, prevent recurrence (complete the treatment plan), work on remaining metacognitive beliefs, predict possible motivators and discuss how to use a new processing program, schedule reinforcement sessions	90

### Ethical considerations

The study has been performed in accordance with the Declaration of Helsinki and has been approved by the Ethics Committee of the Research Deputy of Bushehr University of Medical Sciences with the ethics code [IR.BPUMS.REC.1395.52]. After a brief explanation about the objectives of the study for the participants, written informed consent was obtained from study participants. Confidentiality was ensured.

### Data analysis

Data were analyzed using descriptive statistics (frequency, mean and standard deviation) and inferential statistics via SPSS23 (SPSS, Inc., Chicago, IL, USA) software at a significance value less than 0.05. The distribution of variables was analyzed by the Kolmogorov–Smirnov test at a significant value of 0.05. Since the distributions of the studied variables were not normal, non-parametric statistical tests such as Chi-square for comparison of demographic variable, Mann Whitney for between-group comparison, and Wilcoxon and Friedman tests for within-group comparisons were used to analyze the data.

## Results

**Fig. 1:**
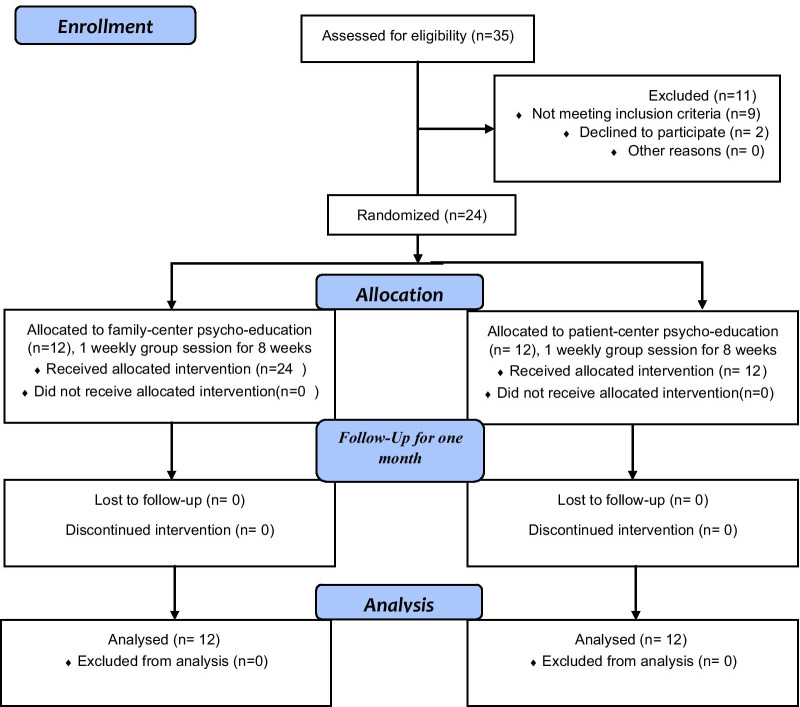
Study flow chart

For each group, the study flowchart displays the number of participants who were randomly assigned, received the intended intervention, and were analyzed for the primary outcome (Fig. 1) according to the CONSORT diagram. The trial was completed on schedule.

The participants were in the range of 27–67 years old with an average of 50.75 (11.34) and 47.33 (12.34) for the control and experimental groups, respectively. The results indicated that the variables, including marital status, occupational status, educational status, place of residence, history of hospitalization, history of mastectomy and chemotherapy, history of psychotherapy in both experimental and control groups were not statistically significant according to the Chi-square test (*p* = 0.05) (Table 2).

**Table 2:** Descriptive characteristics

Variable	Category	Control	Experimental	*p* value
		(n = 12)	(n = 12)	
Marital status (%)	Single	2 (16.7)	1 (8.3)	0.34^a^
	Married	10 (83.3)	11 (91.7)	
Educational status (%)	Non academic	12 (100)	11 (91.7)	0.13^a^
	Academic	0 (0)	1 (8.3)	
Occupation (%)	Housewife	5 (41.7)	7 (58.3)	0.16^a^
	Retired	5 (41.7)	4 (33.34)	
	Employee	2 (16.6)	1 (8.3)	
Place of residence (%)	City	6 (50)	7 (58.3)	0.11^a^
	Village	6 (50)	5 (41.7)	
History of hospitalization (%)	Yes	12 (100)	12 (100)	
	No	0 (0)	0 (0)	
Mastectomy and chemotherapy	Yes	12 (100)	12 (100)	
	No	0 (0)	0 (0)	
History of psychotherapy (%)	Yes	0 (0)	0 (0)	
	No	12 (100)	12 (100)	

^a^_Chi-square test_

In the experimental group and a within-group comparison, results showed a significant difference between the T0 and T1 of depression scores (*p* = 0.025). Moreover, the within-group comparisons revealed that the depression score of the patients in the experimental group in T2 was significantly lower than the depression score in T1 (*p* = 0.019). These changes did not show a significant difference in the control group (*p* = 0.393). Besides, the within-group analysis indicates the difference between the level of depression in T0 and T2 (*p* = 0.016) (Table 2). The results indicated no difference in the mean depression scores between the two groups in T0 (*p* = 0.416). The results also indicated no difference in the mean score of depression between the two groups in T1 (*p* = 0.073), but the depression score in the two groups in T2 showed a significant difference (*p* = 0.006), so that the mean score of depression was lower in the experimental group than in the control group (Table 3).

**Table 3:** Mean difference in primary and secondary outcome at T0 (baseline), T1 (after intervention) and T2 (follow-up) in the experimental/control groups

Variable	Group	T0 M (SD)	T1 M (SD)	*p* value	T2 M (SD)	*p* value
Depression	Experimental	21.16 (4.83)	15.58 (8.50)	0.025^a^	12.83 (88.21)	0.016^b^
	Control	22.66 (5.34)	23.16 (8.89)	0.969^a^	25.75 (8.22)	0 .285^b^
	*p* value	0.416^c^	0.073^c^		0.006^c^	
CE regulation	Experimental	105 (14.72)	108.41 (15.80)	0.442^a^	111.08 (23.30)	0.480^b^
	Control	108 (13.08)	111.58 (11.56)	0.102^a^	110.16 (7.76)	0.656^b^
	*p* value	0.644^c^	0.644^c^		0.908^c^	
M C beliefs	Experimental	79.51 (10.72)	72.66 (11.42)	0.050^a^	68.72 (15.74)	0.006^b^
	Control	76.33 (12.08)	76.01 (11.62)	0.769^a^	77/91 (12.94)	0.906^b^
	*p* value	0.506^c^	0.488^c^		0.088^c^	

^a^Wilcoxon test

^b^Wilcoxon test (compare T0 to T2)

^c^Mann Whitney test

Regarding secondary outcomes, there was no significant difference between the mean score of cognitive-emotional regulation in T1 and T2 between the experimental and control groups (*p* = 0.908) (Table 3). The within-group comparison indicated no difference in the total score of cognitive-emotional regulation in T1 and T2 in both experimental (*p* = 0.758) and control (*p* = 0.627) groups. The comparison revealed no difference in the total score of meta-cognitive beliefs in T0 (*p* = 0.506), T1 (*p* = 0.488), and T2 (*p* = 0.088) in patients participating in two groups. No significant difference was observed between the scores before and immediately after the intervention in the experimental group (*p* = 0.051). However, a significant difference was found between the pre-test and follow-up the periods scores (*p* = 0.006), so that the follow-up score was lower than the pre-test score. No significant difference was found between the scores of meta-cognitive beliefs before, immediately after the intervention and during the follow-up period in the control group (*p* = 0.062) (Table 3).

## Discussion

The purpose of this research was to determine the effectiveness of MCT on depression, cognitive emotions regulation, and meta-cognitive beliefs in depressed women with breast cancer. The results of the post-test response indicated that the average of depression in the experimental group was significantly decreased from the baseline response. These results did not show a significant difference between posttest and one-month follow-up measurements. This can support the long-term effects of MCT as well as the sustainability of the effects on depression in these patients. The results of a clinical trial study were consistent with the results of the present study and showed a reduction in depression in 70% of patients in the post-test and in 90% of the patients in the follow-up period [30]. In addition, in Iran, Ashoury applied two methods of CBT and MCT in his study and concluded that MCT and CBT were more effective than pharmacotherapy alone in the treatment of major depressive disorder [31]. Furthermore, the results of some studies were consistent with the results of this study and reveal a decrease in depression after the intervention of MCT [32–35]. It should be noted that patients are taught in MCT to challenge beliefs using attention-teaching techniques to reduce them, to cut them off by practice or to change their beliefs. Accordingly, the self-regulatory executive function is disrupted in these individuals, and the likelihood of relapse of depression disorder is reduced. In the present study, the rate of depression in the follow-up period was reduced further in comparison to the post-test period. It appears that, since meta-cognitive therapy focuses on mental processes and its controlling factors, changes in these structures require time lapse. In other words, since the thought content is interfered in this therapeutic approach, more time will be required for the emergence of therapeutic effects than therapies targeting behavioral modification.

Other findings of the present study indicated that MCT had no significant effect on cognitive-emotional regulation. The results of a study that expresses the direct relationship between the effect of MCT on the cognitive-emotional regulation of depressed people [36], contrasts with the results of our study, which indicate further studies in this regard. However, it is important to note that cancer has different pressures and effects on affected patients’ lives. These patients also struggle with the stress and complications of cancer treatments and disease-related crises in addition to depression [37]. It appears that the severity of stress, along with depression can reduce the effect of the intervention; this is demonstrated in the form of the lack of cooperation of these patients in doing their assignment, reduction of their motivation, and lack of cooperation with their therapist. Furthermore, scientists explain the effect of aging on the reduction of meta-cognitive perceptual function. The study participants’ average age was relatively high, and it appears that their high mean age affected the reduction of the treatment effect [38].

The data indicate the reduction of metacognitive beliefs scores in the experimental group. A significant reduction was found in metacognitive beliefs scores compared to the pre-test and one-month follow-up scores. Wells et al. (2012) stated that the metacognitive beliefs score in depressed patients was decreased after metacognitive therapy [39]. The finding of some trial studies also exhibited a reduction in the meta-cognitive beliefs score during MCT [40–42].

Non-homogeneity of the group therapy, lack of controlling the severity of the disease in the group, selection of a sample from only one clinic in Shiraz, and short-term follow-up period were the limitations of this research. Thus, the generalization of the trial findings should be done with caution. It is recommended that future studies be conducted with higher standards for sampling as well as inclusion and exclusion criteria to enhance the generalizability. It is also recommended that some studies be conducted with long-term follow-up.

## Conclusion

The results of this study indicated that the MCT was effective in depressed patients with breast cancer and reduced the level of depression and meta-cognitive beliefs in the patients. These results lead us to use new methods of psychotherapy like metacognitive therapy. Women are the mainstay of the family, and their mental health ensures the health of the family and society. Moreover, owing to the increasing rate of breast cancer and its psychological consequences, effective treatment of these consequences will prevent challenges in these people’s lives.

## Supplementary information

**Additional file 1:** Beck Depression Inventory (BDI).

**Additional file 2:** Cognitive Emotion Regulation Questionnaire (CERQ).

**Additional file 3:** Meta-Cognitions Questionnaire (MCQ).

## Data Availability

The datasets used and/or analyzed during the current study are available from the corresponding author on reasonable request.

## Abbreviations

MDD: Major depressive disorder
MCT: Meta cognitive therapy
MCB: Meta cognitive beliefs
MCQ: Meta cognitive questionnaire
CERQ: Cognitive emotional regulation questionnaire
TAU: Treatment as usual
