# Supplementary material for: Group meta-cognitive therapy and depression in women with breast cancer: a randomized controlled trial
Source: BMC Womens Health. 2021 Mar 18;21:111. doi: 10.1186/s12905-021-01258-9 (PMC7977266; doi:10.1186/s12905-021-01258-9)
Supplement: Supplementary file 1 — Additional file 1: Beck Depression Inventory (BDI). [file 12905_2021_1258_MOESM1_ESM.pdf]

Beck's Depression Inventory

This depression inventory can be self-scored. The scoring scale is at the end of the questionnaire.

1.

0 I do not feel sad.
1 I feel sad
2 I am sad all the time and I can't snap out of it.
3 I am so sad and unhappy that I can't stand it.

2.

0 I am not particularly discouraged about the future.
1 I feel discouraged about the future.
2 I feel I have nothing to look forward to.
3 I feel the future is hopeless and that things cannot improve.

3.

0 I do not feel like a failure.
1 I feel I have failed more than the average person.
2 As I look back on my life, all I can see is a lot of failures.
3 I feel I am a complete failure as a person.

4.

0 I get as much satisfaction out of things as I used to.
1 I don't enjoy things the way I used to.
2 I don't get real satisfaction out of anything anymore.
3 I am dissatisfied or bored with everything.

5.

0 I don't feel particularly guilty
1 I feel guilty a good part of the time.
2 I feel quite guilty most of the time.
3 I feel guilty all of the time.

6.

0 I don't feel I am being punished.
1 I feel I may be punished.
2 I expect to be punished.
3 I feel I am being punished.

7.

0 I don't feel disappointed in myself.
1 I am disappointed in myself.
2 I am disgusted with myself.
3 I hate myself.

8.

0 I don't feel I am any worse than anybody else.
1 I am critical of myself for my weaknesses or mistakes.
2 I blame myself all the time for my faults.
3 I blame myself for everything bad that happens.

9.

0 I don't have any thoughts of killing myself.
1 I have thoughts of killing myself, but I would not carry them out.
2 I would like to kill myself.
3 I would kill myself if I had the chance.

10.

0 I don't cry any more than usual.
1 I cry more now than I used to.
2 I cry all the time now.
3 I used to be able to cry, but now I can't cry even though I want to.

11.
0 I am no more irritated by things than I ever was.
1 I am slightly more irritated now than usual.
2 I am quite annoyed or irritated a good deal of the time.
3 I feel irritated all the time.

12.
0 I have not lost interest in other people.
1 I am less interested in other people than I used to be.
2 I have lost most of my interest in other people.
3 I have lost all of my interest in other people.

13.
0 I make decisions about as well as I ever could.
1 I put off making decisions more than I used to.
2 I have greater difficulty in making decisions more than I used to.
3 I can't make decisions at all anymore.

14.
0 I don't feel that I look any worse than I used to.
1 I am worried that I am looking old or unattractive.
2 I feel there are permanent changes in my appearance that make me look unattractive
3 I believe that I look ugly.

15.
0 I can work about as well as before.
1 It takes an extra effort to get started at doing something.
2 I have to push myself very hard to do anything.
3 I can't do any work at all.

16.
0 I can sleep as well as usual.
1 I don't sleep as well as I used to.
2 I wake up 1-2 hours earlier than usual and find it hard to get back to sleep.
3 I wake up several hours earlier than I used to and cannot get back to sleep.

17.
0 I don't get more tired than usual.
1 I get tired more easily than I used to.
2 I get tired from doing almost anything.
3 I am too tired to do anything.

18.
0 My appetite is no worse than usual.
1 My appetite is not as good as it used to be.
2 My appetite is much worse now.
3 I have no appetite at all anymore.

19.
0 I haven't lost much weight, if any, lately.
1 I have lost more than five pounds.
2 I have lost more than ten pounds.
3 I have lost more than fifteen pounds.

20.

0	I am no more worried about my health than usual.
1	I am worried about physical problems like aches, pains, upset stomach, or constipation.
2	I am very worried about physical problems and it's hard to think of much else.
3	I am so worried about my physical problems that I cannot think of anything else.

21.

0	I have not noticed any recent change in my interest in sex.
1	I am less interested in sex than I used to be.
2	I have almost no interest in sex.
3	I have lost interest in sex completely.

INTERPRETING THE BECK DEPRESSION INVENTORY

Now that you have completed the questionnaire, add up the score for each of the twenty-one questions by counting the number to the right of each question you marked. The highest possible total for the whole test would be sixty-three. This would mean you circled number three on all twenty-one questions. Since the lowest possible score for each question is zero, the lowest possible score for the test would be zero. This would mean you circles zero on each question. You can evaluate your depression according to the Table below.

Total Score	Levels of Depression
1-10	These ups and downs are considered normal
11-16	Mild mood disturbance
17-20	Borderline clinical depression
21-30	Moderate depression
31-40	Severe depression
over 40	Extreme depression

http://www.med.navy.mil/sites/NMCP2/PatientServices/SleepClinicLab/Documents/Beck_Depression_Inventory.pdf
