# Supplementary material for: Group meta-cognitive therapy and depression in women with breast cancer: a randomized controlled trial
Source: BMC Womens Health. 2021 Mar 18;21:111. doi: 10.1186/s12905-021-01258-9 (PMC7977266; doi:10.1186/s12905-021-01258-9)
Supplement: Supplementary file 2 — Additional file 2: Cognitive Emotion Regulation Questionnaire (CERQ). [file 12905_2021_1258_MOESM2_ESM.doc]

**CERQ**

**© Garnefski, Kraaij & Spinhoven, 2001**

**How do you cope with events?**

Everyone gets confronted with negative or unpleasant events now and then and everyone responds to them in his or her own way. By the following questions you are asked to indicate what you generally think, when you experience negative or unpleasant events.

|  | **(almost) never** | **some-**  **times** | **regu-larly** | **often** | **(almost) always** |
| --- | --- | --- | --- | --- | --- |
| 1. 1 feel that I am the one to blame for it | 1 | 2 | 3 | 4 | 5 |
| 2. I think that I have to accept that this has happened | 1 | 2 | 3 | 4 | 5 |
| 3. I often think about how I feel about what I have experienced | 1 | 2 | 3 | 4 | 5 |
| 4. I think of nicer things than what I have experienced | 1 | 2 | 3 | 4 | 5 |
| 5. I think of what I can do best | 1 | 2 | 3 | 4 | 5 |
| 6. I think I can learn something from the situation | 1 | 2 | 3 | 4 | 5 |
| 7. I think that it all could have been much worse | 1 | 2 | 3 | 4 | 5 |
| 8. I often think that what I have experienced is much worse than what others have experienced | 1 | 2 | 3 | 4 | 5 |
| 9. I feel that others are to blame for it | 1 | 2 | 3 | 4 | 5 |
| 10. I feel that I am the one who is responsible for what has happened | 1 | 2 | 3 | 4 | 5 |
| 11. I think that I have to accept the situation | 1 | 2 | 3 | 4 | 5 |
| 12. I am preoccupied with what I think and feel about what I have experienced | 1 | 2 | 3 | 4 | 5 |
| 13. I think of pleasant things that have nothing to do with it | 1 | 2 | 3 | 4 | 5 |
| 14. I think about how I can best cope with the situation | 1 | 2 | 3 | 4 | 5 |
| 15. I think that I can become a stronger person as a result of what has happened | 1 | 2 | 3 | 4 | 5 |
| 16. I think that other people go through much worse experiences | 1 | 2 | 3 | 4 | 5 |
| 17. I keep thinking about how terrible it is what I have experienced | 1 | 2 | 3 | 4 | 5 |
| 18. I feel that others are responsible for what has happened | 1 | 2 | 3 | 4 | 5 |
| 19. I think about the mistakes I have made in this matter | 1 | 2 | 3 | 4 | 5 |
| 20. I think that I cannot change anything about it | 1 | 2 | 3 | 4 | 5 |
| 21. I want to understand why I feel the way I do about what I have experienced | 1 | 2 | 3 | 4 | 5 |
| 22. I think of something nice instead of what has happened | 1 | 2 | 3 | 4 | 5 |
| 23. I think about how to change the situation | 1 | 2 | 3 | 4 | 5 |
| 24. I think that the situation also has its positive sides | 1 | 2 | 3 | 4 | 5 |
| 25. I think that it hasn’t been too bad compared to other things | 1 | 2 | 3 | 4 | 5 |
| 26. I often think that what I have experienced is the worst that can happen to a person | 1 | 2 | 3 | 4 | 5 |
| 27. I think about the mistakes others have made in this matter | 1 | 2 | 3 | 4 | 5 |
| 28. I think that basically the cause must lie within myself | 1 | 2 | 3 | 4 | 5 |
| 29. I think that I must learn to live with it | 1 | 2 | 3 | 4 | 5 |
| 30. I dwell upon the feelings the situation has evoked in me | 1 | 2 | 3 | 4 | 5 |
| 31. I think about pleasant experiences | 1 | 2 | 3 | 4 | 5 |
| 32. I think about a plan of what I can do best | 1 | 2 | 3 | 4 | 5 |
| 33. I look for the positive sides to the matter | 1 | 2 | 3 | 4 | 5 |
| 34. I tell myself that there are worse things in life | 1 | 2 | 3 | 4 | 5 |
| 35. I continually think how horrible the situation has been | 1 | 2 | 3 | 4 | 5 |
| 36. I feel that basically the cause lies with others | 1 | 2 | 3 | 4 | 5 |
| **Thank you for filling out the questionnaire!** | | | | | |
