# Supplementary material for: Group meta-cognitive therapy and depression in women with breast cancer: a randomized controlled trial
Source: BMC Womens Health. 2021 Mar 18;21:111. doi: 10.1186/s12905-021-01258-9 (PMC7977266; doi:10.1186/s12905-021-01258-9)
Supplement: Supplementary file 3 — Additional file 3: Meta-Cognitions Questionnaire (MCQ). [file 12905_2021_1258_MOESM3_ESM.pdf]

Metacognition Questionnaire-30 (MCQ-30)

	Items	Do not agree 1	Agree slightly 2	Agree moderately 3	Agree very much 4
**1**	Worrying helps me to avoid problems in the future				
**2**	My worrying is dangerous for me				
**3**	think a lot about my thoughts				
**4**	I could make myself sick with worrying				
**5**	I am aware of the way my mind works when I am thinking through a problem				
**6**	If I did not control a worrying thought, and then it happened, it would be my fault				
**7**	need to worry in order to remain organized				
**8**	I have little confidence in my memory for words and names				
**9**	My worrying thoughts persist, no matter how I try to stop them				
**10**	Worrying helps me to get things sorted out in my mind				
**11**	I cannot ignore my worrying thoughts				
**12**	I monitor my thoughts				
**13**	I should be in control of my thoughts all of the time				
**14**	My memory can mislead me at times				
**15**	My worrying could make me go mad				
**16**	I am constantly aware of my thinking				
**17**	I have a poor memory				
**18**	pay close attention to the way my mind works				
**19**	Worrying helps me cope				
**20**	Not being able to control my thoughts is a sign of weakness				
**21**	When I start worrying, I cannot stop				
**22**	I will be punished for not controlling certain thoughts				
**23**	Worrying help me to solve problems				
**24**	I have little confidence in my memory for places				
**25**	It is bad to think certain thoughts				
**26**	I do not trust my memory				
**27**	If I could not control my thoughts, I would not be able to function				
**28**	I need to worry, in order to work well				
**29**	I have little confidence in my memory for actions				
**30**	I constantly examine my thoughts				

Scoring and Interpretation Information

Subscale scores range from 6 to 24, and total scores range from 30 to 120, with higher scores indicating higher levels of unhelpful metacognitions (for example, high scores on "cognitive confidence" indicate distrust of memory and other unhelpful beliefs about their cognition). Results are also presented as percentiles based on a normative community sample.

Subscales are calculated by summing the following items:

- (Lack of) Cognitive Confidence: 8, 14, 17, 24, 26 and 29

- Positive Beliefs about Worry: 1, 7, 10, 19, 23 and 28

- Cognitive Self-Consciousness: 3, 5, 12, 16, 18 and 30

- Negative Beliefs about Uncontrollability and Danger: 2, 4, 9, 11, 15 and 21

- Need to Control Thoughts: 6, 13, 20, 22, 25 and 27
